# Lens capsule-related complications in cataract phacoemulsification: Advances in pathophysiology and clinical management

**DOI:** 10.1016/j.aopr.2026.05.002

**Published:** 2026-05-14

**Authors:** Qian Luo, Lei Yang, Xiangbin Guan, Furong Liao, Li Jiang, Yumeng Bao, Jingyi Zhu, Yanping Song

**Affiliations:** a Medical College, Wuhan University of Science and Technology, Wuhan, China; b Department of Ophthalmology, General Hospital of Central Theater Command of People's Liberation Army, Wuhan, China

**Keywords:** Cataract, Lens capsule-related complications, Phacoemulsification surgery

## Abstract

**Background:**

Phacoemulsification surgery is one of the most widely performed ophthalmic procedures for cataract treatment worldwide, and the occurrence of lens capsule-related complications has a significant impact on surgical outcomes and visual prognosis of patients.

**Main text:**

In this review article, we focus on lens capsule-related complications, including posterior capsule rupture, anterior capsular tear, capsule bend, anterior and posterior capsule opacification, capsular bag distension syndrome, capsular contraction syndrome, and dead bag syndrome. Drawing on both basic research and clinical evidence, we provide an updated overview of current evidence regarding their underlying mechanisms and key clinical treatment strategies. The risk factors for the occurrence of various complications are explored. The core pathophysiological mechanisms, including changes in capsule biomechanics, as well as the proliferation, migration, and fibrosis of lens epithelial cells, are analyzed in detail.

**Conclusions:**

Based on the above analysis, we summarize prevention and management strategies in hopes of improving the safety and effectiveness of cataract surgery.

## Highlights

• Elucidates lens capsule biomechanics and LECs' pathological roles in lens capsule-related complications.
• Reviews underlying mechanisms for lens capsule-related complications from rupture, capsular bend, ACO, PCO, CBDS to DBS.
• A summary of preventive and management strategies to improve surgical outcomes and visual quality in postoperativepatients.

## 1 Introduction

Cataract is characterized by decreased transparency and increased opacity in the crystalline lens. Approximately 95 million people worldwide are affected, and cataract is the leading cause of reversible visual impairment and blindness worldwide. Currently, the standard treatment for cataracts is surgical removal of the cloudy lens by phacoemulsification followed by implantation of an intraocular lens (IOL).^1^^,^^2^

The crystalline lens is a transparent avascular structure resembling a double convex lens, which is divided into four main layers arranged from outer to inner: the capsule, the epithelial layer, the cortical layer, and the nucleus. The crystalline lens capsule is a specialized basement membrane layer composed primarily of type IV collagen, laminin, and heparan sulfate proteoglycans,^3^^,^^4^ which provides structural support and maintains intraocular stability and serves as a selective permeability barrier regulating the exchange of nutrients (glucose), water, oxygen and waste products (lactate) between the aqueous humor and the lens interior.^5^

Lens epithelial cells (LECs) have the potential for proliferation, differentiation, and migration. Together, they maintain the integrity and stability of the lens in a relatively isolated environment, playing a key role in maintaining long-term transparency.^4^, ^5^, ^6^ Clinically, the capsule is divided into anterior and posterior capsules based on the position relative to the equator. The anterior lens capsule (ALC) membrane is significantly thicker than the posterior lens capsule (PLC) membrane, with maximal thickness in the peripheral area (equatorial region) where the suspensory ligament attaches to the anterior and posterior parts and minimal thickness at the posterior pole.^7^ The ALC basement membrane is acellular and transparent; however, it is lined internally by a single layer of cuboidal LECs, which are the primary source of its extracellular matrix components.^8^^,^^9^ As the thickest basement membrane in the human body, the ALC, together with the PLC, forms an essential barrier.^8^ LECs are a single layer of cuboidal epithelial cells, which are distributed in the anterior subcapsular and equatorial parts of the capsule (without epithelial cells in the PLC). Among them, the cell division in the pre-equatorial and equatorial regions (germinal regions) is active. The cells gradually elongate and extend, assume a columnar shape, and then differentiate into lens fibers. The anterior LECs are the primary source supplying the extracellular matrix (ECM) in the lens capsule, consisting of type IV collagen, laminin, cysteine-rich acidic secretory protein (SPARC), heparan sulfate proteoglycans (HSPGs), and other components.^4^

In phacoemulsification surgery, continuous curvilinear capsulorhexis (CCC) removes the central area of the ALC while preserving the intact PLC and peripheral area of the ALC. This residual capsule structure is called the “pocket”.^10^ After surgery, residual LECs initiate repair and regeneration processes at the pocket opening, with the core event being fibrotic scar formation. This process originates from the destruction of capsule integrity, which exposes LECs to a nonphysiological environment, triggering a series of fibrotic reactions, including epithelial-mesenchymal transition (EMT), and ECM proliferation, deposition, and contraction.^11^, ^12^, ^13^, ^14^ The hydrodissection, phacoemulsification, cortical resorption, and IOL implantation during surgery all constitute disturbances to the capsule microenvironment. Through mechanisms such as mechanical injury, suspensory ligament tension, and the presence of residual LECs and their activation, the pathophysiological initiation point of lens capsule-related complications is established, ultimately compromising surgical effectiveness, visual quality, and prognosis of patients. Although individual complications have been reviewed extensively, in this review, we uniquely integrate their shared pathophysiological mechanisms—specifically LEC behavior, capsular biomechanics, and inflammatory cascades—across the spectrum of capsular complications. By synthesizing mechanistic insights with practical management strategies, we aim to provide clinicians with a unified conceptual framework for risk stratification, intraoperative decision-making, and postoperative surveillance. This integrated approach facilitates recognition of complication interrelationships, such as how anterior capsule polishing affects both anterior capsule opacification (ACO) and regenerative posterior capsule opacification (PCO) risk, and informs personalized prevention strategies.

## 2 Method of literature search

This review concentrates on the latest pathophysiology and clinical management of lens capsule-related complications. A thorough literature search of English and Chinese-language publications was conducted. Databases including PubMed, Web of Science, Google Scholar, China National Knowledge Infrastructure (CNKI) and Wanfang were searched for relevant studies. The final search was performed in December 2025. The search strategy included MeSH terms and keywords such as “lens capsule-related complications,” “capsular-related complications,” “posterior capsule rupture,” “posterior capsule tear,” “posterior capsule rent,” “capsular bend,” “anterior capsule opacification,” “posterior capsule opacification,” “capsular bag distension syndrome,” “capsular contraction syndrome,” “dead bag syndrome,” “phacoemulsification surgery complications,” “cataract surgery complications,” and “lens capsule”. The references of searched articles were also browsed for a comprehensive literature review.

Inclusion criteria were: (1) original research articles (randomized controlled trials, cohort studies, case–control studies, cross-sectional studies, and laboratory investigations), systematic reviews, and meta-analyses; and (2) publications in English or Chinese. Exclusion criteria were: (1) conference abstracts, editorials, commentaries, and letters without original data; and (2) studies not directly addressing lens capsule-related complications. Two independent reviewers (QL and LY) screened titles and abstracts, followed by full-text assessment; disagreements were resolved by discussion or consultation with a third reviewer. The search yielded 1915 records, of which 182 full-text articles were assessed for eligibility, and 100 studies were ultimately included in this synthesis.

## 3 Posterior capsule rupture (PCR) and anterior capsular tear (ACT)

ACT and PCR denote inadvertent breaches of the anterior and posterior lens capsules occurring during cataract surgery, respectively. Epidemiological studies report an overall PCR incidence ranging from 0.2% to 14%, whereas the incidence of ACT during CCC was between 0.79% and 5.55%, underscoring their comparable clinical relevance.^15^, ^16^, ^17^, ^18^, ^19^

Surgical inexperience represents a shared primary risk factor for both complications. Although their risk profiles exhibit partial overlap, they differ in emphasis: ACT risk is predominantly driven by patient-specific factors—particularly inherent capsular fragility (e.g., associated with pseudoexfoliation syndrome, congenital aniridia, or prior ocular trauma) and conditions that heighten surgical complexity (e.g., elevated intralenticular pressure in hypermature cataracts or inadequate pupillary dilation). In contrast, PCR risk is more strongly linked to anatomical challenges—including zonular instability, shallow anterior chamber depth, and small pupil size, as well as increased lens density or complete nuclear opacification.^7^^,^^15^^,^^17^, ^18^, ^19^, ^20^

Regarding pathogenesis, PCR arises from multilevel pathophysiological interactions. Direct mechanical surgical trauma—such as instrument contact, high-vacuum aspiration, or excessive intraocular pressure manipulation—represents a common initiating factor.^15^ An intrinsic structural weakness of the posterior capsule, particularly at the central posterior pole, constitutes the underlying anatomical predisposition. Systemic factors—including advanced age and diabetes—further impair capsular biomechanical integrity by disrupting collagen metabolism (e.g., via advanced glycation end-products (AGE)–mediated cross-linking, upregulation of matrix metalloproteinases (MMPs), and reactive oxygen species (ROS) accumulation) and by compromising LEC function. Following capsular rupture, a cascade of biological responses ensues, characterized by inflammatory cytokine release, amplified oxidative stress, and aberrant LEC proliferation—collectively promoting wound extension and impeding tissue repair.^7^^,^^15^^,^^21^, ^22^, ^23^, ^24^, ^25^, ^26^, ^27^, ^28^, ^29^ In contrast, the mechanism of ACT is more straightforward and predominantly mechanical: it results from discrete intraoperative events, including inadequate control of tangential force during capsulorhexis; instrument entanglement with the capsular margin; a sudden intracapsular pressure surge during hydrodissection; or abnormal mechanical stress on the capsular bag during IOL implantation—all of which may precipitate a linear anterior tear or propagate a preexisting one.^17^^,^^18^^,^^20^ In summary, PCR reflects a complex pathophysiological disequilibrium involving anatomical vulnerability, molecular and cellular alterations, and iatrogenic trauma, whereas ACT is primarily attributable to direct mechanical disruption occurring at specific surgical steps.

In light of these mechanisms, preoperative risk stratification for ACT and PCR is indicated in patients with pseudoexfoliation, Marfan syndrome, coloboma, hypermature/traumatic cataract, lens-induced glaucoma, prior vitrectomy, high myopia, diabetes, small pupil, shallow chamber, or weak zonules.^15^^,^^23^^,^^30^ Intraoperatively, steps that are especially critical include redirecting the tear counterclockwise, avoiding hydrodissection in specific conditions, performing viscodissection/centripetal dragging, layered hydrodissection, low-flow/low-vacuum aspiration, ceasing aspiration when posterior capsule folds appear, and ophthalmic viscoelastic device replenishment before IOL insertion.^15^^,^^18^^,^^30^ Postoperatively, signs such as vitreous prolapse and blurred vision/floaters warrant vigilance for retinal detachment, macular edema, and IOL dislocation, necessitating intensified anti-inflammatory therapy and close monitoring.^7^^,^^15^

Intraoperative management of PCR depends on the stage of occurrence and extent of vitreous loss.^29^ Vitrectomy is needed to clear vitreous from the anterior chamber and remove nuclear fragments from the vitreous. IOL placement depends on residual capsular support; when capsular support permits, a single-piece IOL is implanted in the bag; for inadequate support, a three-piece IOL is placed in the sulcus or scleral fixation is performed.^7^^,^^15^^,^^23^ For anterior capsule tears, converting small radial tears (<3 clock hours) to a curvilinear configuration with microforceps is recommended.^31^ Low-flow phacoemulsification (bottle height <80 cm, vacuum <250 mmHg) minimizes capsular stress.^18^ In-the-bag IOL with optic capture is preferred when capsular support is adequate; sulcus or scleral-fixated IOLs are used for extensive compromise (≥6 clock hours).^32^

## 4 Capsular bend

In modern phacoemulsification surgery, the capsular bag–IOL complex forms after the IOL is implanted into the capsular bag, and then the ALC and PLC adhere to the edge of the IOL to form a barrier.^33^ Adhesion progresses from the peripheral capsule towards the optic edge of the IOL, and ultimately, the PLC wraps around the edge of the IOL to form the capsular bend. The occurrence of capsular bend is influenced by the axial length of the eye, the capsule size, the integrity and centering of the capsulorhexis during surgery, and the size of the ALC opening.^34^

The formation of capsule adhesion begins in the early postoperative period (0–1 week). During this time, the aqueous humor infiltrates and causes the contracted ALC membrane to adhere tightly to the edge of the IOL, forming initial adhesion points. Residual LECs begin to secrete ECM to promote biological healing (Fig. 1A). Subsequently (at 2–4 weeks), the contraction force of the ALC and the radial tension of the IOL loop reach equilibrium, pushing the edge of the ALC opening towards the PLC, causing peripheral adhesion. Eventually, the adhesion zone expands toward the center, pulling the PLC forward and bending it by the ALC. A circular ridge-like structure is formed at the posterior optic edge of the IOL (Fig. 1A).^35^

**Fig. 1:**
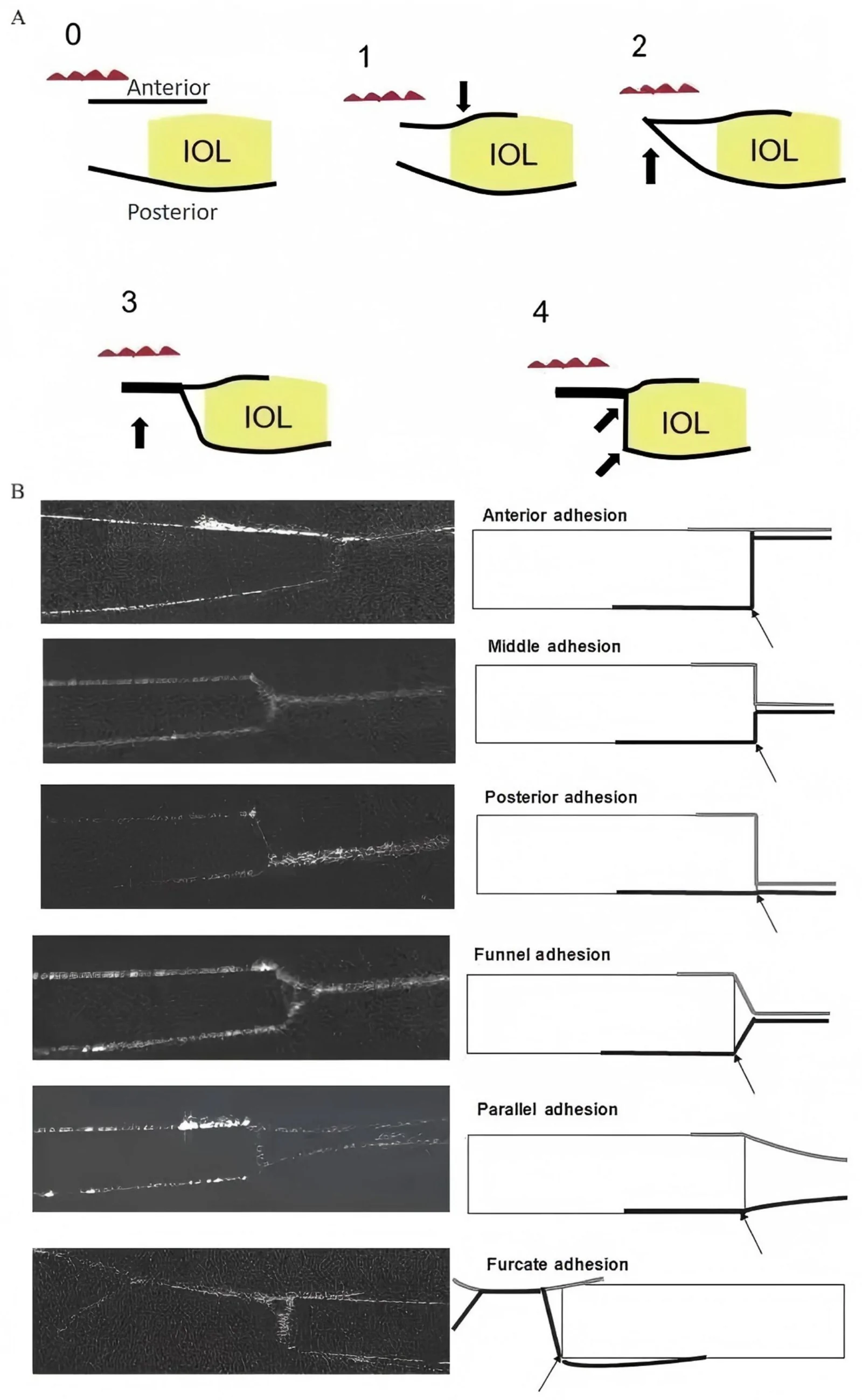
The formation and types of capsular bend. A: Developmental stages (0–4) of the capsular bend based on anterior and posterior capsule attachment. B: Classification of capsular bend types (anterior adhesion type, middle adhesion type, posterior adhesion type, funnel adhesion type, parallel adhesion type and furcate adhesion type).^35^

Based on the morphological relationship between the ALC and PLC membranes and the IOL, the capsular bend can be defined as either complete capsule adhesion (360° coverage of the IOL optical area) or incomplete adhesion. Fully adhesive capsular bend can significantly inhibit LECs migration, effectively reduce the incidence of PCO, and enhance IOL stability.^36^ Two theoretical mechanisms have been proposed to explain this inhibitory effect: cell contact inhibition and mechanical compression. Cell contact inhibition, supported by histopathological research, suggests that the contact inhibition behavior of LECs after contact with the IOL edge hinders their migration. Mechanical compression, supported by anatomical observations of cadaveric eyes and clinical studies of IOL designs with different edges, theorizes that the critical factor is physical obstruction of LECs migration by the contact pressure generated between the IOL edge and the PLC. Research also suggests that fully adhesive capsule bending has a better inhibitory effect due to its ability to create stronger contact pressure.^37^

In contrast, an incomplete adhesion-type capsular bend is prone to PCO and IOL displacement. Capsular bend is the result of the combined effects of cellular, mechanical, and surgical factors. Incomplete LECs removal during surgery increases the risk and severity of incomplete adhesion-type capsule bending. As residual LECs migrate and proliferate to the PLC under the stimulation of inflammation and other factors, cell clusters are formed below the IOL optical surface, and pulling of the capsule promotes bending. Simultaneously, inflammatory reactions caused by surgical trauma promote cytokine release, which in turn promotes LECs proliferation, transdifferentiation, and ECM production, leading to capsular fibrosis and subsequently contributing to the formation of the incomplete adhesion-type of capsular bend.^35^^,^^38^, ^39^, ^40^

A study reported six capsular bend types in a rabbit model: anterior adhesion, middle adhesion, posterior adhesion, funnel adhesion, furcate adhesion, and the parallel adhesion type (Fig. 1 B).^35^ It demonstrated that complete overlap between the anterior capsule and the IOL optic promoted stable capsular bend configurations and significantly delayed the onset of PCO. In contrast, incomplete overlap led to the evolution of the capsular bend into unstable forms, which was associated with an earlier occurrence and higher incidence of PCO.^41^ IOL edge design is crucial, with rectangular edges effectively blocking the backward movement of LECs, facilitating the formation of fully adhesive bending, whereas circular edges are less effective. Weak or dysfunctional suspensory ligaments can cause uneven contraction or displacement of the PLC, leading to incomplete adhesive bending.^40^^,^^42^^,^^43^ During surgery, a CCC of appropriate size can ensure complete coverage of the IOL optical area by the surrounding capsule membrane, promoting stable and fully adhesive bending formation. Conversely, irregular, eccentric, or undersized capsulorhexis can cause asymmetric contraction of the pouch, disrupting bending stability.^42^^,^^44^^,^^45^

Dynamic changes in the capsular bend directly affect the axial position of the IOL, which in turn affects postoperative refractive stability. In clinical practice, the capsule bend index (CBI) is used to evaluate capsule bend formation in different parts of the eye. At the same time, postoperative anterior chamber depth (PAD) reflects changes in the axial optical position of the IOL. Research has shown that when the CBI is <2.3, anterior displacement of the IOL (PAD decrease) leads to myopia drift, whereas when the CBI is >2.3, posterior displacement of the IOL (PAD increase) leads to hyperopia drift. This mechanical mechanism evolves: in the early surgical period, the ALC adhesion dominates and causes the IOL to move forward (myopic tendency); later, enhanced PLC adhesion causes the IOL to move backward (hyperopic tendency). Certain populations are more susceptible to these changes. Individuals with high axial myopia exhibit a postoperative hyperopia shift. In patients with a shallow anterior chamber, postoperative PAD deepening may occur, accompanied by posterior displacement, also manifesting as a hyperopia drift.^38^^,^^39^

These dynamics necessitate preoperative risk stratification for abnormal capsular bend in patients with high axial myopia, shallow anterior chamber, zonular weakness, or those at risk for irregular, eccentric, or undersized capsulorhexis.^34^^,^^37^ Intraoperatively, the critical step is performing a continuous, central, and appropriately sized capsulorhexis to ensure complete IOL optic coverage and promote stable, fully adhesive bending. Postoperative vigilance is required for signs such as unexplained myopic or hyperopic drift, incomplete capsular bend configurations, reduced capsulorhexis diameter, IOL decentration/tilt/rotation, or CBI deviation (<2.3 or >2.3), which warrant close monitoring of CBI and anterior chamber depth during the first 3 months, particularly in high-risk patients.^34^^,^^37^

Clinical management of capsular bend primarily involves recognition and refractive optimization. Ensure complete 360° anterior capsule-IOL optic overlap during surgery to promote complete capsular bend formation and reduce PCO risk. In zonular weakness or high axial length, capsular tension ring (CTR) implantation may enhance capsular stability and promote symmetric bend formation.^46^, ^47^, ^48^

## 5 Anterior capsule opacification (ACO) and posterior capsule opacification (PCO)

Opacification of the lens capsule can appear in the ALC or PLC after cataract surgery. The incidence of PCO within 5 years after surgery ranges from 3% to 50%, whereas ACO occurs less frequently and significantly earlier, sometimes within 1 month after surgery.^49^^,^^50^ Although some factors influencing ACO and PCO overlap, they do differ; both are influenced by material and design characteristics of IOL as well as individual patient factors, including ocular comorbidities. ACO is primarily dependent on the initial diameter of CCC, whereas intraoperative technical factors and postoperative inflammatory reactions also influence PCO.^10^^,^^48^^,^^50^, ^51^, ^52^

ACO and PCO involve the proliferation, metaplasia, and ECM deposition of postoperative LECs, driven by cytokines such as transforming growth factor-β (TGF-β). However, significant differences exist between the two in key cell sources, dominant pathological processes, and histological manifestations.^48^^,^^51^^,^^53^ ACO primarily originates from residual cuboidal epithelial cells (A cells) beneath the ALC. Following surgery, these cells undergo metaplasia, proliferation, and transformation. Although most maintain a single-layer stable structure, some differentiate into contractile myofibroblasts under cytokine stimulation (especially TGF-β), leading to ALC shrinkage and fibrosis. This myofibroblast transformation process drives the differentiation and fibrosis, with histological manifestations characterized by early cell proliferation and late ECM deposition between the ALC membrane and the anterior surface of the IOL optic part (i.e., anterior subcapsular opacification).^48^^,^^50^^,^^53^^,^^54^ PCO, in contrast, involves two types of residual LECs in the anterior (A cells) and equatorial (E cells) regions. Cells transform into myofibroblasts through TGF-β/Smad pathway-mediated EMT, resulting in fibrotic PCO, characterized by matrix deposition and PLC retraction. E cells, in contrast, abnormally differentiate into lens fiber-like cells under stimuli such as basic fibroblast growth factor (bFGF). This process forms regenerative PCO characterized by Soemmerring rings and Elschnig pearls, with Elschnig pearls exerting a more significant impact on vision.^48^^,^^51^^,^^53^, ^54^, ^55^ Given these mechanisms, preoperative risk stratification for ACO and PCO should identify patients with pseudoexfoliation, diabetes, uveitis, high myopia, young age, prior vitrectomy, or complicated cataract surgery. Additional ACO risk arises from a small capsulorhexis and IOL designs that promote capsular contact, whereas residual cortex, intense postoperative inflammation, and hydrophilic IOL materials heighten PCO risk.^10^^,^^48^^,^^49^ Intraoperatively, critical steps include performing a continuous, circular, appropriately sized capsulorhexis; precise in-the-bag implantation; and thorough cortex removal. Postoperatively, unexplained visual decline, capsulorhexis shrinkage to <4 mm, visually significant PCO, and IOL decentration/tilt warrant close monitoring, as ACO typically manifests within 1 month, whereas PCO develops over a period of 3–5 years.^48^^,^^53^

Nd:YAG laser incision remains the primary treatment method for both ACO and PCO. In special cases such as complicated capsular bag distension syndrome (CBDS), infection, severe PCO, or IOL calcification, personalized surgical intervention (such as drainage and IOL replacement) is required. Long-term follow-up shows that IOLs with square optic edges can effectively reduce the need for postoperative Nd:YAG laser treatment.^48^

At the prevention level, the core strategies for ACO and PCO focus on precise surgical techniques, including performing a standardized CCC to achieve a continuous and circular capsulorhexis, selecting square optic edge IOLs to use biological barrier effects in inhibiting LEC migration, precise in-the-bag implantation of IOLs combined with thorough removal of the cortex to limit LEC proliferation space.^10^^,^^48^^,^^50^^,^^55^^,^^56^ However, differences in surgical details between the two exist, especially regarding the application of ALC polishing, which remains controversial. Proponents believe that thorough polishing of the ALC can remove residual LECs and cortical material, effectively inhibiting ACO, delaying capsule fibrosis, significantly reducing the risk of ACO, and potentially inhibiting fibrotic PCO.^57^^,^^58^ Opponents highlight that although ALC polishing may not substantially affect fibrous PCO, it may significantly increase regenerative PCO, which is more harmful to vision. In vitro experiments have further confirmed that the proliferation of residual LECs may be enhanced after polishing.^59^, ^60^, ^61^, ^62^ Therefore, current PCO prevention strategies typically avoid ALC polishing, favoring techniques such as PLC polishing, PLC button surgery, or intraoperative IOL rotation to enhance protection of the PLC.^57^^,^^63^

Drug-based interventions also vary. Established clinical measures include conventional non-steroidal anti-inflammatory drugs (NSAIDs), which act through indirect inhibition of IL-1/IL-6 and are commonly used postoperatively, as well as perioperative corticosteroids.^64^, ^65^, ^66^ Investigational strategies comprise adjunctive antimetabolic drugs (e.g., mitomycin C, 5-fluorouracil) and targeted cytokines (e.g., anti-TGF-β/FGF) for multidimensional synergistic prevention,^51^^,^^67^ together with intraoperative or perioperative immunotoxins and arginine-glycine-asparagine peptide-based inhibitors.^48^^,^^50^^,^^54^ Additional emerging modalities, such as photodynamic therapy and drug-eluting IOL coatings, are under investigation, and all of these approaches require further clinical validation.^68^ Future research focused on the development of targeted drugs and risk prediction models is needed for further optimizing long-term prognosis.

## 6 Capsular bag distension syndrome (CBDS)

CBDS is characterized by CCC of the capsule edge and adhesion of the IOL inside the capsule. This adhesion impedes free fluid flow through the ALC opening, causing fluid accumulation inside the capsule and axial displacement of the IOL, ultimately resulting in decreased visual acuity.^69^^,^^70^ The postoperative incidence of this complication is estimated to be between 0.73% and 1.0%.^71^^,^^72^ Known risk factors for CBDS include axial myopia (long axial length), deep anterior chamber preoperatively, small CCC, phacoemulsification combined with glaucoma surgery, the material and design of the IOL, excessive hydrodissection during surgery, and residual viscoelastic agents.^70^ According to the time of onset, Miyake classified CBDS into intraoperative, early postoperative, and late postoperative types.^73^

Intraoperative CBDS primarily results from excessive hydrodissection, which causes accumulation of balanced salt solution in the capsule. This pushes the lens nucleus forward, causing excessive expansion of the PLC. Early postoperative CBDS often arises from incomplete clearance of the posterior viscoelastic agent around the IOL, leading to anterior IOL displacement, myopic drift, a shallow anterior chamber, and increased intraocular pressure. Late-stage CBDS may be asymptomatic and is only discovered when the patient perceives a visual impairment.^70^^,^^71^

The pathogenesis of CBDS is multifactorial. First, an osmotic pressure effect can occur when residual LECs and their protein by-products and fragments, residual lens cortex fragments, and viscoelastic agents (such as sodium hyaluronate) accumulate in the capsule, leading to an osmotic gradient that draws aqueous humor into the capsule. Analytical studies (e.g., Western blotting for proteins) have reported that concentrations of α-crystallin and β-crystallin in the turbid fluid are higher than in the aqueous humor. Additionally, calcium (Ca^2+^) levels are also elevated. However, Sugiura et al. analyzed the extracted liquid using high-performance liquid chromatography and confirmed the presence of sodium hyaluronate.^74^, ^75^, ^76^ Second, adhesion between the edge of the ALC and the optical zone of the IOL may occur. This phenomenon may be related to the formation of an inflammatory fibrin membrane on the surface of the IOL due to postoperative inflammation, which results in a partially closed cavity. Third, infection with *Propionibacterium* *acnes* has been implicated. Recent studies have reported that the presence of *Propionibacterium* *acnes* in aspirated fluid may cause low-toxicity endophthalmitis. However, it remains unknown whether this strain is a pathogenic factor or a chance discovery.^70^^,^^77^^,^^78^ Fourth, elevated levels of inflammatory factors, such as interleukin-8 (IL-8) and tumor necrosis factor-α (TNF-α) in the cystic fluid indicate that local inflammatory reactions may exacerbate fluid accumulation.^79^ Fifth, rapid eye movements, such as saccades or horizontal nystagmus, can generate rotational forces, increasing the pressure inside the capsule and promoting fluid infiltration.^80^ Other contributing factors include implanting the IOL in an inverted orientation, which may lead to capsular bag occlusion, and special surgical techniques such as envelope resection, which can lead to reclosure of the capsular pocket.^69^^,^^70^^,^^81^^,^^82^

In managing CBDS, the risk factors detailed above identify the patients who warrant preoperative stratification. Intraoperatively, critical steps include avoiding a capsulorhexis diameter <5 mm, meticulous removal of retrolenticular viscoelastic, limiting hydrodissection, and selecting IOLs that minimize forward displacement and capsular adhesion.^67^^,^^69^^,^^70^ Postoperatively, a shallow anterior chamber, elevated intraocular pressure, myopic shift, and progressive blurred vision warrant immediate evaluation. Slit-lamp evidence of anterior IOL displacement with fluid accumulation and anterior segment optical coherence tomography (OCT) or ultrasound biomicroscopy facilitate early detection.^67^^,^^80^^,^^81^

Early postoperative CBDS is usually managed conservatively using topical corticosteroids. Nd:YAG laser posterior capsulotomy, which is safe, effective, and can improve refractive errors, is the preferred treatment method. Compared with laser, pars plana vitrectomy combined with posterior capsulotomy allows more thorough removal of effusion and turbidity and significantly reduces the risk of endophthalmitis. Other exploratory techniques include pars plana puncture with antibiotic lavage, lateral incision puncture, and scleral tunnel lavage.^69^^,^^74^^,^^83^^,^^84^ Some reports describe loosening the adhesion between the artificial lens and the ALC and flushing the capsule using a perfusion suction head.^74^^,^^85^ In the absence of an Nd:YAG laser, Mardelli described a needle capsulotomy technique using a 30-G needle under a slit lamp to gently push the artificial lens posteriorly to alleviate CBDS.^86^ The use of femtosecond laser-assisted PLC incision for cases where the PLC cannot be clearly visualized was also reported.^87^

Effective prevention and treatment of CBDS require a balance of intraoperative precision and individualized postoperative management. Future strategies should focus on technological innovations, such as intelligent IOL design and intraoperative monitoring, as well as the improvement of standardized guidelines to reduce CBDS incidence and adverse prognoses. IOL design is a key preventive and controllable factor. Early studies report that its specific materials and structural features, including soft, large-size loops, biconvex optics, and hydrophobic acrylate loops can increase the risk by promoting forward IOL movement, increasing the contact with the ALC, and forming a closed space.^70^

## 7 Capsular contraction syndrome (CCS)

CCS, first described by Davison, is characterized by reduction of the capsulorhexis area and the equatorial diameter of the lens capsule, ACO, and IOL deviation and tilt.^88^ The incidence of CCS ranges between 1.4% and 14.0%, most commonly happened between 3 and 30 weeks after surgery. Contraction peaks within the first month after surgery and stabilizes within 3 months.^46^

The primary risk factor is surgical technique, particularly the diameter of the capsulorhexis during surgery and incomplete polishing of the ALC. In addition, the design and type of IOL are relevant, as well as the presence of systemic and ocular diseases.^46^^,^^89^ CCS fundamentally represents the cascade reaction of residual LECs after surgery, driven by the pathological microenvironment resulting from surgical trauma, IOL stimulation, inflammatory factors, and the disruption of the blood–aqueous and blood–retinal barriers. Abnormal activation of factors such as TGF-β2 initiates EMT, transforming the cell morphology from cuboidal epithelia to spindle-shaped, fibroblast-like cells, fundamentally reshaping the molecular phenotype. This is accompanied by downregulation of E-cadherin and upregulation of transcription factors such as α-smooth muscle actin, Snai1, Twist, and zinc finger E-box binding homeobox 1,and these cells then secrete excessive ECM, such as collagen type I, IV, V, VI and fibronectin and acquire strong contractile ability, depositing and cross-linking between the ALC of the lens and the optical zone of the IOL, forming dense fibrotic plaques or opaque membranes, and leading to thickening and stiffness of the capsule.^12^^,^^46^

This pathological process is centrally regulated by the TGF-β/Smad pathway and co-amplified by the mitogen-activated protein kinase / phosphoinositide 3-kinase–protein kinase B (MAPK/PI3K–AKT) pathway, inflammatory cascade (such as IL-1β/NF-κB, TNF-α), the Wnt/β-catenin pathway, mechanical stress, oxidative stress (ROS/RhoA/ROCK), and growth factor signaling networks (such as PDGF).^12^ Mechanical stress is a key driver unique to CCS. The centripetal force generated by the contraction of lens fibers at the capsulorhexis edge is higher than the centrifugal force generated by normal lens fiber contraction, resulting in a biomechanical imbalance of the capsule and progressive shrinkage of the ALC opening. Capsule instability, which is caused by weak suspensory ligaments and physical stimulation of the IOL, transmits mechanical signals through the cytoskeleton, inhibits the Hippo pathway, and activates the transcription co-activator YAP/TAZ, which enters the nucleus. After binding to TEAD, EMT and fibrosis genes can be directly activated. Simultaneously, integrins sense changes in ECM hardness and activate focal adhesion kinase (FAK)/Src, which in turn triggers the extracellular signal-regulated kinase/PI3K–AKT pathway, converting mechanical signals into biochemical responses and converging with TGF-β and growth factor signals.^90^, ^91^, ^92^

Conditions such as diabetes and myotonic dystrophy increase the risk of CCS, as do certain IOL materials (e.g., hydrophobic acrylate IOL), which can form a physical barrier between the ALC and the PLC due to their good bioadhesion with the capsule, effectively inhibiting LECs migration and the EMT process. However, owing to their poor adhesion, hydrophilic acrylic or poly(methyl methacrylate) IOLs are more likely to allow LECs to proliferate, migrate, and deposit ECM in the optical zone.^46^^,^^89^^,^^90^

Preoperative risk stratification should explicitly identify patients with diabetes, high myopia, retinitis pigmentosa, uveitis, advanced age, glaucoma, or myotonic dystrophy.^45^^,^^88^ Intraoperatively, critical steps include controlling the capsulorhexis diameter at 5.0–5.5 mm; thoroughly polishing the anterior capsular inner surface to remove residual LECs; selecting a hydrophobic acrylic IOL with a plate-haptic, multi-haptic, or square-edge design; and, in high-risk patients, implanting a CTR to distribute capsular stress.^45^^,^^46^^,^^88^ Postoperatively, glare, blurred vision, hyperopic shift, a measured anterior capsular opening <4 mm, or IOL decentration warrant immediate evaluation. Ultrasound biomicroscopy or OCT can quantify capsular shrinkage and fibrotic thickness.^46^^,^^88^

Prevention of CCS focuses on optimizing surgical technique, including controlling the diameter of the capsulorhexis, thoroughly polishing the ALC, selecting appropriate IOL, and implementing targeted interventions for high-risk patients. After surgery, close monitoring of the capsule opening, IOL position, and visual acuity is required, alongside standardized anti-inflammatory measures. When the ALC is severely contracted (≤4 mm) or the IOL is displaced, Nd:YAG laser radial/annular ALC incision or femtosecond laser release is used. In refractory or severely fibrotic cases, surgical intervention is required. Mild to moderate cases may benefit from mechanical dilation of the capsule, which is accompanied by IOL dislocation and requires complex fixation. Severe cases may require capsulotomy, viscoelastic separation, or IOL replacement, with particular attention paid to correcting the possible hyperopic drift caused by CCS.^46^^,^^47^^,^^93^

## 8 Dead bag syndrome (DBS)

DBS is a rare and poorly understood complication of cataract surgery, characterized by degenerative changes in the lens capsule that appear many years after surgery. The epidemiological factors associated with DBS remain unclear. Although pseudoexfoliation syndrome, uveitis, myopia, history of vitrectomy, and factors related to weakened suspensory ligaments, such as eye trauma, have been implicated, none are unique to this syndrome.^94^ Recent evidence suggests a possible genetic susceptibility.^95^ Given its rarity, the specific incidence rate across different genders and age groups has not yet been determined.^94^ The key pathological features of DBS include significant thinning, increased transparency, and relaxation of the capsular pocket, with a distinctive delamination (splitting or stripping) at the suspensory ligament attachment site, leading to loss of structural integrity.^96^ A key finding was the absence of LECs on the inner surface of the pocket, with no evidence of secondary proliferation, fibrosis, or metaplasia. The etiology of LECs loss remains controversial. It may result from primary or secondary degeneration of LECs (involving congenital defects, surgical trauma, over polishing, changes in intra-capsular osmotic pressure such as intumescence cataract, or metabolic/ischemic factors that affect ECM secretion and pocket maintenance), or from abnormalities of the basement membrane of the pocket itself that impair LECs' attachment, survival, and function. This results in a vicious cycle of LECs loss or dysfunction and capsular pocket structure degeneration, which eventually hinders LECs repair.^97^^,^^98^

Histopathological and immunohistochemical analyses (detection of vimentin, collagen I, fibronectin, and collagen IV) have shown that LECs exist transiently and secrete ECM before dying or shedding. In contrast, basement membrane components (collagen IV and laminin) are relatively preserved. However, the stronger immunoreactivity of type IV collagen in the outer capsule may be related to capsule separation.^96^ Delamination at the suspensory ligament attachment point leads directly to progressive weakening of the suspensory ligament support function or even loss of suspensory ligament fibers, which in turn causes delayed subluxation or dislocation of the IOL within the relaxed sac.^99^

DBS is distinct from other causes of lens subluxation, such as pseudoexfoliation syndrome, which often has weak suspensory ligaments and characteristic substance deposition. In contrast, DBS lacks evidence of capsule fibrosis or opacity. Although similarities with true-exfoliation syndrome in terms of capsule stratification exist, the histopathological manifestations are unique. The association between surgical procedures (such as LEC removal or capsular polishing) and the etiology of DBS has not been established. This is evidenced by numerous reported cases that progressed to DBS despite no removal of LECs or polishing. Clearly, the polishing could not completely remove or reach the equatorial LECs. Similarly, DBS has no clear association with specific IOL designs or materials. Overall, the precise triggering factors and detailed pathophysiological processes of DBS still require further research and clarification.^96^, ^97^, ^98^, ^99^, ^100^

From a clinical management perspective, preoperative risk stratification for DBS should identify patients with pre-existing capsular degeneration, pseudoexfoliation syndrome, prior vitreoretinal surgery, or traumatic zonular weakness.^94^ In high-risk eyes, the intraoperative critical step may involve prophylactic fixation of the IOL haptics within the capsular bag (intracapsular suture loop fixation) to prevent late IOL dislocation in the event of progressive capsular degeneration, thereby securing the haptic–capsule complex and mitigating future risk.^100^ Postoperatively, unexplained intraocular pressure elevation, recurrent visual decline, or new-onset flashes and floaters warrant immediate evaluation for secondary glaucoma or rhegmatogenous retinal detachment.^99^

After DBS-induced IOL dislocation, mild dislocation can be observed and monitored, whereas significant dislocation affecting vision requires surgical intervention. Surgical procedure selection depends on the degree of dislocation and the integrity of the capsule. As degenerated pouches cannot provide effective support, the recurrence rate of simple IOL reduction surgery is high. Therefore, IOL replacement surgery combined with scleral suture fixation is recommended to enhance stability, with anterior vitrectomy performed if necessary. The existing innovative intra-pocket suture fixation technology, in which a 9-0 polypropylene suture is used to make Hoffman pockets inside the capsular pocket and fix the IOL dual loops in a loop form, can avoid exposure of the loops and deformation of the tear opening caused by ciliary groove fixation, making it especially suitable for designing IOL with square-edged loops.^100^ Postoperative visual acuity is usually significantly improved, although vigilance for complications such as secondary glaucoma and retinal detachment is required. Long-term efficacy depends on stable IOL fixation rather than on degeneration of the capsule.^99^

The current diagnosis and treatment of DBS relies heavily on empirical surgery, which has significant shortcomings in prevention, early diagnosis, effective treatment, and overall management. In the future, there is an urgent need to integrate basic and clinical research, elucidate the underlying mechanisms more deeply, and fill data gaps exists.

## 9 Conclusions

Although the clinical manifestations of lens capsule-related complications after phacoemulsification surgery vary, common core mechanisms for their occurrence exist: surgical trauma, abnormal proliferation and transformation of residual LECs, and biomechanical changes in the capsule. Among these, the abnormal activity of residual LECs is the most critical. However, each complication has distinct features (Table 1). ACT and PCR are typically acute intraoperative mechanical injuries that directly compromises intraocular stability and visual prognosis (Fig. 2). The capsular bend mainly reflects the influence of biomechanical remodeling of the capsule or changes in IOL position on refraction (Fig. 2). ACO and PCO, particularly PCO, both represent optical interference caused by the proliferation, migration, and metaplasia of residual LECs into fibrous tissue (Soemmering ring) or Elschnig pearls beneath the capsule (Fig. 2). CBDS arises from increased intracapsular osmotic pressure, leading to capsular pocket kyphosis or equatorial bulging (Fig. 2). In contrast, CCS is caused by fibrosis contracture of the ALC, along with excessive contraction of the LECs at the equator, leading to constriction and deformation of the capsular pocket (Fig. 2). Both CBDS and CCS are chronic and progressive morphological abnormalities. The relatively rare but severe DBS is characterized by the progressive degeneration of the pocket, with the attachment of the suspensory ligament to the division of layers, resulting in a loss of structural integrity (Fig. 2).

**Table 1:** Summary of lens capsule-related complications in cataract phacoemulsification surgery.

Complication	Definition	Timing	Major risk factors	Core mechanisms	Prevention	Treatment strategies
PCR	Inadvertent posterior capsule breach during cataract surgery	Intraoperative	Surgical inexperience; anatomical challenges (zonular instability, shallow ACD, small pupil, dense cataract); advanced age; diabetes	mechanical trauma; intrinsic posterior weakness; systemic collagen disruption (AGEs, MMPs, ROS); biological cascade	Preoperative risk stratification; Meticulous technique (avoid high vacuum and excessive IOP, proper phaco parameters, viscoelastic protection)	Vitrectomy; alternative IOL fixation
ACT	Inadvertent anterior capsule breach during cataract surgery	Intraoperative	Surgical inexperience; capsular fragility (pseudoexfoliation, aniridia, trauma); hypermature cataract; poor dilation	Mechanical disruption	Controlled capsulorhexis technique	Small radial tears (<3 h): convert to curvilinear; in-bag IOL with capture if adequate; sulcus/scleral for ≥6 h; low flow phaco
Capsular bend	Capsule-IOL adhesion configuration	Early postoperative (0–4 weeks)	IOL design; CCC size; axial length; LEC removal;	Cellular adhesion + biomechanical remodeling	Optimized CCC; complete LEC removal; rectangular-edge IOL	Complete adhesion: none needed; Incomplete adhesion/severe contraction: YAG laser posterior/anterior capsulotomy; IOL exchange for malposition
ACO	Opacification of anterior lens capsule after cataract surgery	Early postoperative (as early as 1 month)	Small CCC diameter (primary factor); IOL material/design; patient comorbidities	Residual A cells → myofibroblast transformation (TGF-β) → ALC shrinkage, fibrosis; ECM deposition	Standardized CCC; precise in-the-bag implantation; antimetabolics or anti-TGF-β/FGF; ALC polishing controversial	Nd:YAG anterior capsulotomy
PCO	Opacification of posterior lens capsule after cataract surgery	Within 5 years (incidence 3%–50%)	IOL design; patient factors; surgical technique; postoperative inflammation	Fibrotic PCO: EMT (TGF-β/Smad) → myofibroblasts → matrix deposition, PLC retraction. Regenerative PCO: E cells → lens fiber-like cells (bFGF) → Soemmerring rings, Elschnig pearls	Standardized CCC; rectangular-edge IOL; precise in-the-bag implantation; cortex removal; antimetabolics; avoid ALC polishing; PLC polishing	Nd:YAG laser posterior capsulotomy; adjunctive NSAIDs (indirect IL-1/IL-6 inhibition)
CBDS	Fluid accumulation inside capsule	Intraoperative, early postoperative, or late postoperative	Viscoelastic residue; axial myopia, deep ACD, small CCC, IOL design, excessive hydrodissection	Osmotic gradient (LEC by-products, crystallins, viscoelastic); mechanical sealing; *Propionibacterium acnes*; elevated IL-8/TNF-α; rapid eye movements	Avoid excessive hydrodissection; complete viscoelastic removal; select appropriate IOL	Early: topical corticosteroids. Preferred: Nd:YAG capsulotomy; surgical drainage
CCS	Capsular opening contraction	3–30 weeks postoperatively (peak at 1 month, stabilize by 3 months)	Small capsulorhexis; ALC polishing; IOL type; patient comorbidities (etc. diabetes, myotonic dystrophy)	TGF-β2-induced EMT; ECM overproduction; Hippo-YAP/TAZ; integrin-FAK/Src pathways	Optimize capsulorhexis size; ALC polishing; select appropriate IOL; high-risk targeted interventions;	Nd:YAG radial/annular ALC or femtosecond laser; surgical release
DBS	Late capsular bag degeneration with IOL dislocation	Many years postoperatively (late)	Pseudoexfoliation; possible genetic susceptibility; uveitis; myopia; vitrectomy history; trauma; none unique	LEC loss (degeneration or basement membrane defect); capsule degeneration; progressive zonular weakness	minimal capsular trauma and avoidance of over-polishing may be considered (no proven association)	Mild: observe; significant IOL dislocation: scleral suture fixation, intra-pocket suture fixation (9-0 polypropylene)

PCR: posterior capsule rupture; ACD: anterior chamber depth; AGEs: advanced glycation end-product–mediated; MMPs: matrix metalloproteinases; ROS: reactive oxygen species; IOP: intraocular pressure; IOL: intraocular lens; ACT: anterior capsular tear; CCC: continuous curvilinear capsulorhexis; LEC: lens epithelial cell; ACO: anterior capsule opacification; TGF-β: transforming growth factor-β; ALC: anterior lens capsule; ECM: extracellular matrix; PCO: posterior capsule opacification; EMT: epithelial-mesenchymal transition; FGF: fibroblast growth factor; bFGF: basic fibroblast growth factor; NSAIDs: non-steroidal anti-inflammatory drugs; CBDS: capsular bag distension syndrome; IL-8: interleukin-8; TNF-α: Tumor Necrosis Factor-α; CCS: capsular contraction syndrome; TGF-β2: transforming growth factor-β2; DBS: Dead bag syndrome; IL-1: interleukin-1; IL-6: interleukin-6.

**Fig. 2:**
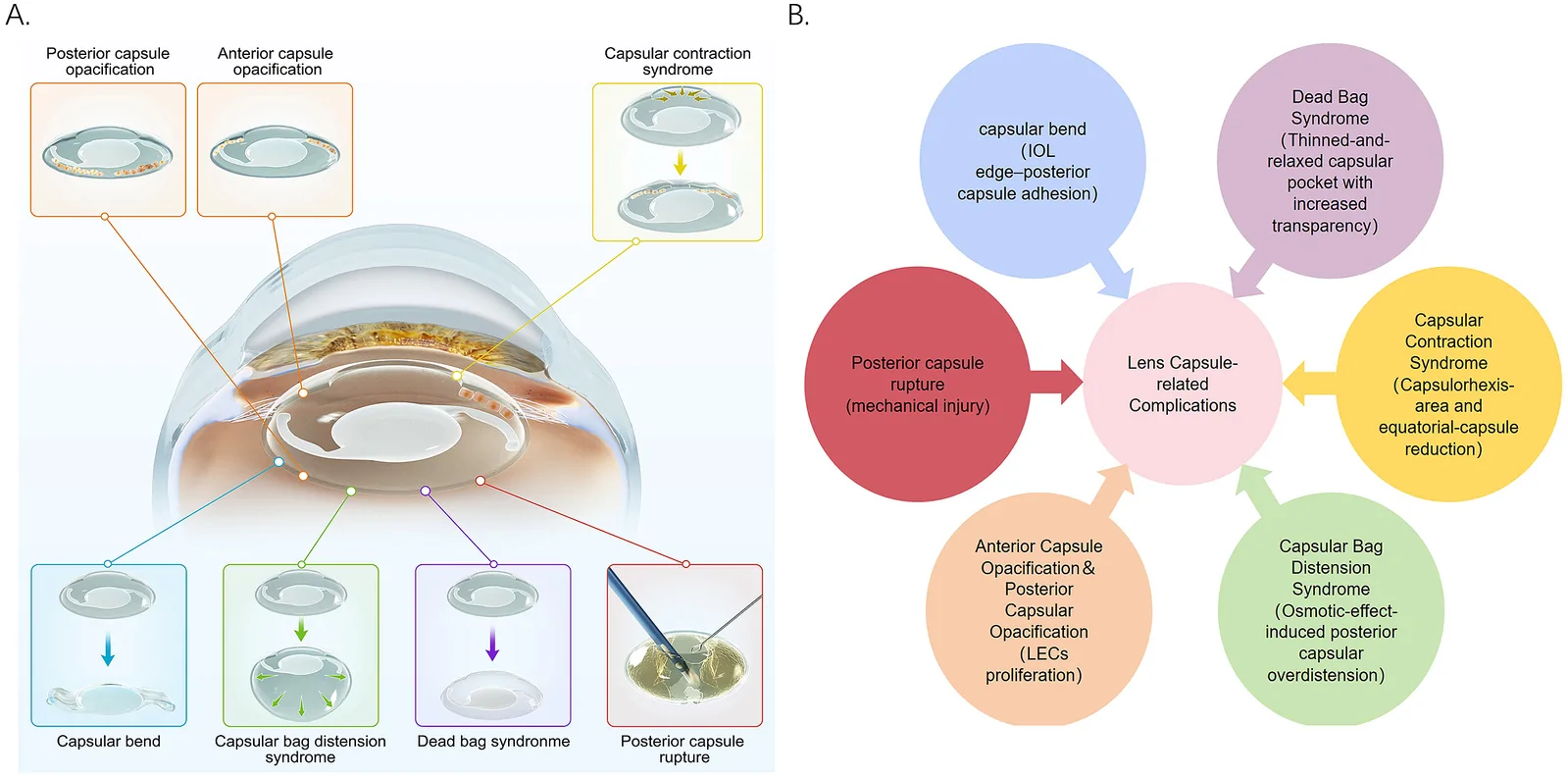
Schematic illustration of the key features and core mechanisms of lens capsule-related complications. (A) Schematic diagram depicting the key features and localization of lens capsule-related complications: Brown: ACO & PCO: Post-cataract surgery opacification localized to the ALC orPLC; Yellow: Capsular Contraction Syndrome: anterior capsulorhexis reduction, equatorial capsular shrinkage, ACO, and IOL tilt/decentration; Blue: Capsular Bend: Adhesion of the ALC and PLC to the intraocular lens edge forms a sealed barrier; Green: Capsular Bag Distension Syndrome: IOL displacement and intracapsular fluid accumulation; Purple: Dead Bag Syndrome: Capsular thinning and transparency; Red: Posterior Capsule Rupture: Rupture or tear of the posterior lens capsule. (B) Core mechanisms of lens capsule-related complications. ACO: anterior capsule opacification; PCO: posterior capsule opacification; ALC: anterior lens capsule; IOL: intraocular lens.

This review has limitations. We synthesize evidence from heterogeneous study designs, and the discussion of rarer complications (e.g., DBS) is necessarily based on lower levels of evidence such as case reports and series. Although we aimed to provide a comprehensive overview, the recommendations should be interpreted within the context of evolving clinical evidence. Notably, this review specifically addresses complications following phacoemulsification for adult age-related cataract, whereas pediatric cataract, traumatic cataract, and other etiologies may present distinct pathophysiological considerations and may require further exploration.

Future management strategies require multidimensional integration. The investigation of underlying mechanisms should integrate molecular biology, materials science, and advanced imaging technologies to identify effective therapeutic targets. In terms of clinical translation, research should focus on developing anti-proliferative and anti-fibrotic agents based on novel targets, as well as exploring their prevention through approaches such as intraocular injection, topical eye drops, IOL surface coating, or intracapsular implants. In terms of individualized management, identification of specific biomarkers for high-risk groups will help guide surgical planning and postoperative management. In terms of surgical techniques, research should focus on developing more minimally invasive and precise procedures, optimizing capsulorhexis (accuracy, symmetry, and centralization) and IOL intracapsular position stability, and reducing complications such as refractive drift. Exploring these complications in depth will promote research on the molecular biology, biomechanics, and intraocular microenvironment of the crystalline lens, which is of great significance for preventing and treating reversible visual impairment worldwide.

## Study approval

Not Applicable.

## Author contributions

The authors confirm contribution to the paper as follows: Conceptualization: JZ, YS; Original draft: QL, LY; Review & editing: LY, XG, FL, L J, XG, YB,YS, JZ; Funding acquisition: JZ.

## Declaration of generative AI use

In preparing this article, ChatGPT was employed solely for polishing language, grammar. Following its use, the authors carefully reviewed and edited the manuscript, and they assume complete responsibility for the final published work.

## Funding

This study was supported by the 10.13039/501100001809National Natural Science Foundation of China (82201191) and the 10.13039/501100003819Natural Science Foundation of Hubei Province (2023AFB461).

## Declaration of competing interest

The authors declare that they have no known competing financial interests or personal relationships that could have appeared to influence the work reported in this paper.
